# Synergistic antioxidant effects of natural compounds on H_2_O_2_-induced cytotoxicity of human monocytes

**DOI:** 10.3389/fphar.2022.830323

**Published:** 2022-09-01

**Authors:** Reda Ben Mrid, Najat Bouchmaa, Wessal Ouedrhiri, Abdelhamid Ennoury, Zakia ZouaouI, Imad Kabach, Mohamed Nhiri, Rachid El Fatimy

**Affiliations:** ^1^ Institute of Biological Sciences (ISSB-P), Mohammed VI Polytechnic University (UM6P), Ben-Guerir, Morocco; ^2^ Laoratory of Inorganic Chemistry, Department of Chemistry, University of Helsinki, Helsinki, Finland; ^3^ Laboratory of Biochemistry and Molecular Genetics, Faculty of Science and Technology, Abdelmalek Essaadi University, Tangier, Morocco

**Keywords:** H_2_O_2_-induced cytotoxicity, monocytes, synergistic interaction, antioxidants, ROS protection

## Abstract

Natural compounds are endowed with a broad spectrum of biological activities, including protection against Toxins. Most of them are known for their antioxidant and radical scavenging activities. However, the synergistic combination of these natural molecules is not well studied. Therefore, the present study aims first to investigate the effect of four potent natural molecules [rosmarinic acid (Ros-A), ellagic acid (Ella-A), curcumin (Cur), and syringic acid (Syr-A)] on H_2_O_2_ -induced cell cytotoxicity and oxidative stress on the human monocytes (THP-1) and then to evaluate their combined action effect. Optimal combinations of these molecules were predicted using an augmented mixture design approach. In the first, as preliminary antioxidant activities screening, two *in vitro* assays were adopted to assess the single radicals scavenging activity of these natural compounds, DPPH• and ABTS• + tests. Based on the results obtained, the multitude of optimal formulas proposed by the mixture design study led to choosing four potent compositions (comp) in addition to ellagic acid, proposed as the most efficient when applied alone. The different molecules and mixtures were used to assess their cytoprotective effect on THP-1 cells in the presence and absence of H_2_O_2_. The most potent Comp-4, as well as the molecules forming this mixture, were exploited in a second experiment, aiming to understand the effect on oxidative stress *via* antioxidant enzyme activities analysis in the H_2_O_2_-induced oxidative stress in the THP-1 cell line. Interestingly, the natural molecules used for THP-1 cells treatment exhibited a significant increase in the antioxidant defense and glyoxalase system as well as suppression of ROS generation evaluated as MDA content. These results indicate that the natural compounds tested here, especially the synergistic effect of Cur and Ros-A (Comp-4), could serve as cytoprotective and immunostimulant agents against H_2_O_2_-induced cytotoxicity THP-1 cells, which makes them interesting for further investigations on the molecular mechanisms in preclinical animal models.

## 1 Introduction

A complex biological response, oxidative stress results from irritation caused by chemical or biological elements such as infections and toxins (Bastin et al., 2021). Cell oxidative stress is mainly related to an imbalance between the increase in reactive oxygen species (ROS) and/or the decrease in the antioxidant defense system efficiency (Bouchmaa et al., 2018a; Xue et al., 2020).

During oxidative stress, ROS are produced as byproducts of oxygen metabolism. The three most well-known ROS are the superoxide anion (O_2_•-), the hydroxyl radical (HO•), and hydrogen peroxide (H_2_O_2_). Hydrogen peroxide is assumed to be responsible for generating intracellular hydroxyl radicals. However, these free radicals are permanently neutralized in healthy cells by enzymatic and non-enzymatic antioxidant systems (Kim et al., 2018; Liu et al., 2019).

Several studies have addressed the intracellular generation of ROS by THP-1 cells (Reddi and Tetaliet, 2019; Bastin et al., 2021); therefore, these cells constitute a good model for studying the effect of toxins and/or for the discovery and development of new anti-oxidative stress compounds.

Different scientists have been interested in the chemo-preventive ability of natural compounds, mainly phytochemicals, to alleviate oxidative stress. Among the different classes of natural compounds, polyphenols and flavonoids are the most evaluated as antioxidants. For instance, ellagic acid is a potent antioxidant compound for which the activity could be directly due to its ability to scavenge free radicals or regulate antioxidant enzyme activities (Marković et al., 2013; Amirahmadi et al., 2021). Curcumin (Cur) is another well-known polyphenol extracted from turmeric (Curcuma longa Linn.), which is known for its wide range of biological activities, including the antioxidant effect (Miao et al., 2015). Curcumin has been reported to inhibit lipid peroxidation and reduce levels of hydroxyl radicals.

Moreover, it was reported that curcumin could reduce lipid peroxidation in renal epithelial cells and protect them from the cytotoxic effect of H_2_O_2_ (Motterlini et al., 2000; Ahangarpour et al., 2019). Rosmarinic acid (Ros-A) was also reported to protect the effect against oxidative and inflammatory stresses. Indeed, in a rat model of neuropathic pain, Ros-A provided a protective effect through its antioxidant and anti-inflammatory activities (Rahbardar et al., 2018).

The synergistic effect of natural compounds has also become a trend in this field, and many studies highlighted the use of mixture design as a powerful statistical and mathematical tool (Ouedrhiri et al., 2016), where the proportions of the components under inquiry are the independent variables in the mixture design, which is a type of response surface experiment. Only the proportions of the mixture components affect the dependent variable called response. The goal of the mixture design approach is to develop better or more inventive formulations and build theoretical constructs regarding reactions and interactions between independent components.

In the present study, the human monocytes (THP-1) cells were exploited as a model to assess the cytoprotective effect of 5 natural compounds and their mixtures on H_2_O_2_-induced oxidative stress. Furthermore, the effect on oxidative stress and the antioxidant enzyme activities was also evaluated to determine the possible molecular mechanisms of these molecules to prevent H_2_O_2_-oxidative damage.

## 2 Materials and methods

### 2.1 Chemical

All the natural compounds tested in this study were purchased from Sigma Aldrich, and their purity was as indicated by the supplier (Cur ≥ 80, Syringic acid ≥ 95%, Ella-A ≥ 95%, Ros-A ≥ 98%, Sinapic acid ≥ 98%, Vanillic acid ≥ 97%, Artemisinin ≥ 98%, Caryophyllene oxide ≥ 99%, Diosgenin ≥ 99%, Naringenin ≥ 95%, berberine ≥ 99%, and Kaempferide ≥ 95%).

### 2.2 Antioxidant activities

#### 2.2.1 ABTS+ radical scavenging activity

The ABTS+ free radical scavenging activity was estimated according to Re et al. (1999) method. ABTS+ was generated by the oxidation of ABTS with potassium persulfate. First, the stock solution of ABTS+ was diluted with methanol to obtain an absorbance of 0.700 ± 0.020 at 734 nm. Then 75 μl of appropriately diluted extracts were added to 925 μl of the diluted ABTS+ solution, and the absorbance was measured at 734 nm after 10 min incubation at room temperature. The free radical scavenging activity was calculated using the following equation:

% Scavenging effect=[(A0−A1)/A0]×100

Where $A_{1}$ is the absorbance value of the sample, and $A_{0}$ is the absorbance of the ABTS+ solution. Scavenging activity in this assay was expressed by IC_50,_ which corresponds to the sample required to inhibit 50% of the ABTS+ Scavenging activity.

#### 2.2.2 DPPH radical scavenging assay

The ability to scavenge the DPPH (2,2-diphenyl-1-picrylhydrazyl) Radical of molecules was measured using the method described by Hatano et al. (1988) with some modifications. First, Extract solutions (50 μl) were mixed with freshly prepared DPPH solution (150 μl). Then, a vigorous mixture was shaken and incubated at room temperature in the dark for 30 min. Finally, the absorbance of the mixture was measured at 517 nm. The DPPH scavenging activity was determined by calculating the percentage of DPPH discoloration using the following equation:

% Scavenging effect=[(A0−A1)/A0]×100

Where A1 is the absorbance value of the sample solution, and $A_{0}$ is the absorbance of the control reaction. Scavenging activity in this assay was expressed by $IC_{\mathrm{50}}$ , which corresponds to the sample required to inhibit 50% of the DPPH scavenging activity.

### 2.3 Mixture design and statistical analysis

The present study was carried out to promote the development of effective synergistic antioxidant and antitumor formulations. As tools, the mixture design approach was employed.

Polyphenols ternary formulation was based on the simplex-centroid mixture design. This design was realized without constraints and with randomization. The different experiments realized followed the matrix presented in Supplementary Table S1, where the Eqs 1–3 correspond to the pure molecules, Eqs 4–6 correspond to the mixtures half-half of two molecules, and the mixture containing one-third of each molecule present Eq. 7, (Supplementary Table S1).

The antioxidant efficiency of each formula was evaluated using DPPH• and ABTS•+ tests as described above. Afterward, the data were fitted to a special cubic polynomial model applying the least-squares regression to estimate the unknown coefficients in Eq. 1:

Y=b_1X_1+b_2X_2+b_3X_3+b_12X_1X_2+b_13X_1X_3+b_23X_2X_3+b_123X_1X_2X_3 (1)

Where Y is the response ( $IC_{\mathrm{50}}$ ), bi is the magnitude of the effect of each component Xi, bij is the magnitude of the interactive effect of two components, and bijk is the magnitude of the interactive effect of the three components on the response. Xi denotes the proportions of the component (i) in the mixture. This analysis was carried out using SAS JMP software, version 16.

Three mixtures were carried out. Where the Cur and ellagic acid (Ella-A) was used as x1 and x2 in each mixture, and Ros-A, syringic acid (Syr-A), and synapic acid (Syn-A) were used as x3 in M1, M2, and M3, respectively (Supplementary Table S1).

### 2.4 Cytotoxicity

#### 2.4.1 Cell culture and treatment

The human monocytic leukemia (THP-1) cell line was used in this study was cultured in RPMI 1640 culture medium supplemented with 2% L-Glu, 10% of Fetal Calf Serum (FCS), and 1% of antibiotics containing penicillin and streptomycin. Cells were maintained at 37°C in a humidified atmosphere containing 5% CO_2_.

#### 2.4.2 MTT assay for cell viability

The human monocyte THP-1 was used as a model to investigate the *in vitro* antiproliferative/Cytotoxic activity, of four natural compounds alone or combined, using the MTT assay for 48 h as described in our previous works (Bouchmaa et al., 2018b). The human monocytes cells were harvested from starting cultures at the exponential growth phase. The harvested cells were plated at a density of (5 × 10^5^ cells per well) in flat-bottomed 96-well microplates containing 100 μl of complete medium for overnight incubation before treatment. The cells were treated with different concentrations of the different compounds in the presence or not of 200 μM H_2_O_2_. All, the tested molecules (Ros-A, Cur, Syr-A, Ella-A) as well as Comps (M1 to M4) were dissolved in dimethyl sulfoxide (DMSO) completed with the medium. The final concentration of DMSO did not exceed 0.001%. Control cells were treated with DMSO alone (0.001%).

### 2.5 Malondialdehyde content determination

Lipid peroxidation measured as Malondialdehyde (MDA) content in THP-1 cells was determined using thiobarbituric acid (TBA) according to the method described previously (Ohkawa et al., 1979; Kabach et al., 2019) with minor modifications. In brief, cell homogenates, under the conditions described above, were mixed with TCA (20%) and TBA (0.67%). The mixture was heated at 95°C for 1 h. After cooling, 1 ml of n-butanol was added to the mixture followed by centrifugation at 12,000 g for 10 min. Organic supernatant was collected to measure the absorbance at 532 nm.

### 2.6 Determination of the free thiol group

The free thiol concentration of samples was measured using Ellman’s assay (Ellman, 1959). Briefly, 250 μl of samples were incubated with 750 μl of 0.5 mM DTNB in 50 mM K-P buffer, pH 8.0, for 15 min at 37°C, and then the absorbance was read at a wavelength of 410 nm. The free thiol concentration of samples was calculated based on the standard curve prepared by using various concentrations of L-cysteine.

### 2.7 Enzyme activity assays

#### 2.7.1 Preparation of cell extracts for antioxidant enzyme assays

The human THP-1 monocytes cells were treated with: Cur (6.25 or 12.5 μM), Ros-A (25 or 50 μM), Ella (25 or 50 μM), M-4 (12.5 or 25 μM), and 200 μM of H_2_O_2_ for 48 h 200 μM of H_2_O_2_ alone was considered as a negative control. After washing once with PBS (10 mM, pH 7.4), the cells were harvested and centrifuged at 1,200 g for 10 min. The pellet was suspended in 500 μl of lysis buffer composed of 50 mM Tris-HCl, 1 mM phenylme-thanesulfonyl (PMSF), 0.1% (v/v) Triton X-100, in 1.5 ml Eppendorf tubes and maintained in constant agitation at 4°C for 30 min. The homogenate was then centrifuged (1,600 g, 20 min) at 4°C. The supernatant (enzyme extract solution) was kept at −80°C or used for the determination of Superoxide dismutase (SOD), Glutathione Peroxidase (GPx), Catalase (CAT), Thioredoxin reductase (TrxR), glutathione reductase (GR), Isocitrate dehydrogenase (NADP + -ICDH) activities and Glyoxalase system (Gly I, II).

#### 2.7.2 Antioxidant enzyme assays

The total SOD activity was assayed according to the method of Sun et al. (1988) with some modifications. Briefly, the reaction mixture contained 0.05 M phosphate buffer, pH 7.5, 10 mM methionine, 0.1 μM EDTA, 2 μM riboflavin, 75 μM Nitro Blue Tetrazolium (NBT), and the enzyme extract. The SOD activity was measured at 560 nm. One unit of SOD activity was defined as the quantity of SOD required to obtain a 50% inhibition of the reduction of NBT. The activity was expressed as units per mg of protein content.

The total GPx activity was measured by the method of Lawrence and Burk (1976) with some modifications. The reaction mixture contained 0.1 M potassium phosphate, pH 7.0, 1 mM EDTA, 1 mM sodium azide, 1 mM GSH, GR (10 μg/ml), 0.25 mM NADPH and enzyme extract. The mixture was incubated at 25°C for 3 min and completed by adding 0.25 mM of H_2_O_2_. The rate of NADPH oxidation was monitored at 340 nm for 5 min. GPx activity was calculated and expressed as nmol of NADPH oxidized/min/mg protein by using the extinction coefficient of 6.2 mM^−1^cm^−1^.

The total TrxR was measured as the reduction of DTNB (5,5′-dithiobis (2-nitrobenzoic acid)) in the presence of NADPH (Lim et al., 2005). The reaction mixture contained 0.1 M phosphate buffer, pH 7.6, 1 mM EDTA, 0.25 mM NADPH, 1 mM DTNB and enzyme extract. The increase in the absorbance at 412 nm was monitored at 25°C. TrxR activity was expressed as nmol of DTNB reduced/min/mg protein by using the extinction coefficient of 13.6 mM^−1^cm^−1^.

The total GR activity was estimated by a modified method by Carlberg and Mannervik (1975). Briefly, the reaction mixture contained 0.1 M phosphate buffer, pH 7.6, 1 mM GSSG, 0.2 mM NADPH. The contents were incubated at 25°C for 3 min, and the reaction was initiated by adding enzyme extract. The rate of NADPH oxidation was monitored at 340 nm. GR activity was expressed as nmol of NADPH oxidized/min/mg protein by using the extinction coefficient of 6.2 mM^−1^cm^−1^.

NADP + -ICDH activity was estimated according to the procedure of Leterrier et al. (2007).

Catalase activity was determined according to the protocol described by El Omari et al. (2018).

Glyoxalase I (EC: 4.4.1.5) assay was carried out according to Hasanuzzaman and Fujita, (2011) with some modifications. Briefly, the assay mixture contained 50 mM K-P buffer (pH 6.6), 2 mM GSH and 2 mM methylglyoxal (MG) in a final volume of 1 ml. The addition of MG started the reaction, and the increase in absorbance was recorded at 240 nm for 5 min. The activity was calculated using the extinction coefficient of 3.37 mM^−1^ cm^−1^.

Glyoxalase II (EC: 3.1.2.6) activity was determined according to the method of Principato et al. (1987) with minor modifications by monitoring the formation of GSH at 412 nm for 5 min. The reaction mixture contained 50 mM Tris–HCl buffer (pH 7.2), 0.2 mM DTNB, and 1 mM S-D—lactoylglutathione (SLG) in a final volume of 1 ml. The addition of SLG started the reaction, and the activity was calculated using the extinction coefficient of 13.6 mM^−1^ cm^−1^ (Ben Mrid et al., 2018).

#### 2.7.3 Protein content determination

The total protein content of the samples was determined following the method of Brad-ford (Bradford, 1976), using BSA as a protein standard.

### 2.8 Statistical approaches

Data were subjected to one-way ANOVA, and differences were determined by Tukey’s multiple comparison test with GraphPad Prism 9.0.2 (134) software for macOS. Each experiment was repeated at least three times. Data are the means of individual experiments and presented as mean ± standard deviation (SD); *p* < 0.05 was considered statistically significant.

## 3 Results

### 3.1 Antioxidant activity of the selected molecules

The molecules tested in the present study were selected based on their importance as natural compounds endowed with several biological activities, mainly antioxidant and anti-inflammatory (Table 1). From our first screening of a list of 12 natural compounds, which showed differentially antioxidant activity (Table 1), five were retained as they showed an enjoyable antioxidant activity. The structures of these compounds are shown in Supplementary Figure S1. Indeed, two *in vitro* assays were used to assess the antioxidant activity of these natural compounds, DPPH• and ABTS•+ tests. The results for the five selected molecules are shown in Table 1. The different compounds IC_50_ of DPPH radical scavenging activities varied from 48.03 to 96.73 µM. Ros-A showed the highest ability to scavenge DPPH• free radicals (IC_50_ = 48.03 μM), followed by Ella (48.35 μM), Cur (59.8 μM), Syr (90.39 μM), and Syn (96.73 μM). Regarding the ABTS assay, Ella exhibited the highest activity with an IC_50_ of 27.69 μM, followed by Ros-A (77.45 μM), Syn-A (146.97 μM), Syr-A (160.77 μM), and Cur (170.38 μM).

**TABLE 1:** The *in vitro* antioxidants activities of natural molecules (Cur, Ros-A, Ella-A, Syr-A, and Syn-A) using DPPH and ABTS assays. The activity was expressed by IC_50_ and data presented as mean ± SD (*n* > 3). Different letters indicate significant differences among treatment at *p* < 0.05.

	IC_50_ (µM)		
Molecule	DPPH	ABTs	References
Curcumin	59.80 ± 2.91	170.38 ± 0.48	Miao et al. (2015)
Syringic acid	90.39 ± 1.40	160.77 ± 3.08	Vo et al. (2020)
Ellagic acid	48.35 ± 2.16	27.6 9 ± 0.81	Marković et al. (2013), Amirahmadi et al. (2021)
Rosmarinic acid	48.03 ± 3.74	77.45 ± 0.048	Rahbardar et al. (2018)
Sinapic acid	96.73 ± 1.06	146.97 ± 1.78	Nićiforović and Abramovic, (2014)
Vanillic acid	2529.61 ± 207.88	>1 mM	Vinothiya and Ashokkumar, (2017)
Artemisinin	2875.17 ± 180.52	>1 mM	Panda et al. (2019)
Caryophyllene oxide	1163.82 ± 230.85	>1 mM	Coté et al. (2017), Nuzul Hakimi Salleh et al. (2015)
Diosgenin	2772.545 ± 220.25	>1 mM	Selim and Al Jaouni, (2016)
Naringenin	2591.84 ± 221.15	>1 mM	Cavia-Saiz et al. (2010)
Berberine	1157.286 ± 280.51	>1 mM	Li et al. (2014)
Kaempferide	2066.20 ± 260.30	>1 mM	Ma et al. (2021)

### 3.2 Validation and establishment of response prediction models

Six models (M) have been established using the Simplex mixture design approach, describing the variations of IC_50_ in ABTS+ and DPPH tests scavenging for the three mixtures. The analysis of variance (ANOVA) showed that the main effect of linear regression is significant since the probability of the significance of the risk *p*-value is less than 0.05 for each mixture. Values of coefficient of determination R^2^ also showed the significative correlation between the experimental values and those predicted by the mathematical model. Results that confirm statistical and mathematical model validation was presented.

As mentioned before, all the tested phenolic compounds exhibited significant antioxidant activity in both tests (ABTS and DPPH). However, Ella-A and Ros-A were the most efficient. Estimated magnitudes highlighted many binary and ternary synergistic antioxidant effects between these compounds. In general, a negative sign of a coefficient in fitted models implies that its related factor has the power to reduce the response (synergistic effect), while a positive sign suggests that the factor can increase the response variable (antagonistic effect).

ABTS•+ test underlined Cur and Ros-A in mixture M1, Ella-A and Syr-A acids in mixture M2, and Cur and Syn-A in mixture M3. While ternary synergy was exhibited only in mixtures M1 and M3.

DPPH scavenging revealed that all binary interactions were significantly synergistic in M1. Besides, Ella-A showed a synergistic interaction with curcumin and Syr-A in M2 and Syn-A in M3. However, the ternary interaction induced a significant antagonistic outcome in M1 and M2.

In summary, the three models obtained for ABTS•+ and DPPH tests are in Eqs 2–7, respectively:

Y=170.38X_Cur+27.69X_(Ella−A)+77.45X_(Ros−A)+261.88X_CurX_(Ella−A)+38.02X_CurX_(Ros−A)+207.39X_(Ella−A)X_(Ros−A)−2941.31X_CurX_(Ella−A)X_(Ros−A) (2)

Y=170.38X_Cur+27.69X_(Ella−A)+160.76X_(Syr−A)+261.88X_CurX_(Ella−A)+68.95X_CurX_(Syr−A)−209.71X_(Ella−A)X_(Syr−A)−2277.146X_CurX_(Ella−A)X_(Syr−A) (3)

Y=170.38X_Cur+27.69X_(Ella−A)+146.96X_(Syr−A)+261.88X_CurX_(Ella−A)−309.74X_CurX_(Syr−A)−1433.184X_CurX_(Ella−A)X_(Syr−A) (4)

Y=59.8X_Cur+48.34X_(Ella−A)+48.02X_(Ros−A)−46.25X_CurX_(Ella−A)−101.69X_CurX_(Ros−A)−44.97X_(Ella−A)X_(Ros−A)+687.31X_CurX_(Ella−A)X_(Ros−A) (5)

Y=59.8X_Cur+48.34X_(Ella−A)+90.39X_(Syr−A)−46.25X_CurX_(Ella−A)+121.49X_CurX_(Syr−A)−145.51X_CurX_(Ella−A)X_(Syr−A)−61.93X_CurX_(Ella−A)X_(Syr−A) (6)

Y=59.8X_Cur+48.34X_(Ella−A)+96.73X_(Syr−A)−81.21X_CurX_(Syr−A)+1578.27X_CurX_(Ella−A)X_(Syr−A) (7)

### 3.3 Optimal formulation

The M2 polyphenols formulated the low IC50 obtained in both tests with a binary interaction between Ella-A and Syr-A. By IC_50_ of 19.28 and 23.08 μM for ABTS and DPPH tests, respectively, the formulations greatly exceed the single antioxidant effect (Supplementary Figure S2). In comparison, the M1 mixture exhibits by its ternary optimum the same antioxidant activity as the M3 mixture presenting only Ella-A as an efficient formula by IC_50_ of 26.55 and 38.37 μM in ABTS and DPPH tests, respectively. Otherwise, M1 presented an optimal binary mixture with an IC_50_ of 22.07 μM in the DPPH test.

Based on these results, the multitude of optimal formulas proposed by the mixture design study led to choosing four compositions (comp) presented in Table 2 to evaluate their cytotoxicity against THP-1 cells, and ellagic acid was proposed as the most efficient when applied alone. Hence, the effect of Ella-A and the individual and mixed effects of the molecules forming the four mixtures were evaluated.

**TABLE 2:** Compounds ratio of optimal compositions.

	Cur	Ella-A	Ros-A	Syr-A	Syn-A
Comp-1	—	0.815	—	0.183	—
Comp-2	—	0.631	—	0.369	—
Comp-3	0.279	0.333	0.388	—	—
Comp-4	0.362	—	0.638	—	—

Cur, Curcumin; Ella-A, ellagic acid; Ros-A; Syr-A, syringic acid; Syn-A, sinapic acid.

### 3.4 Cytotoxic activity of the different natural compounds and the most efficient compositions on THP-cells

**FIGURE 1:**
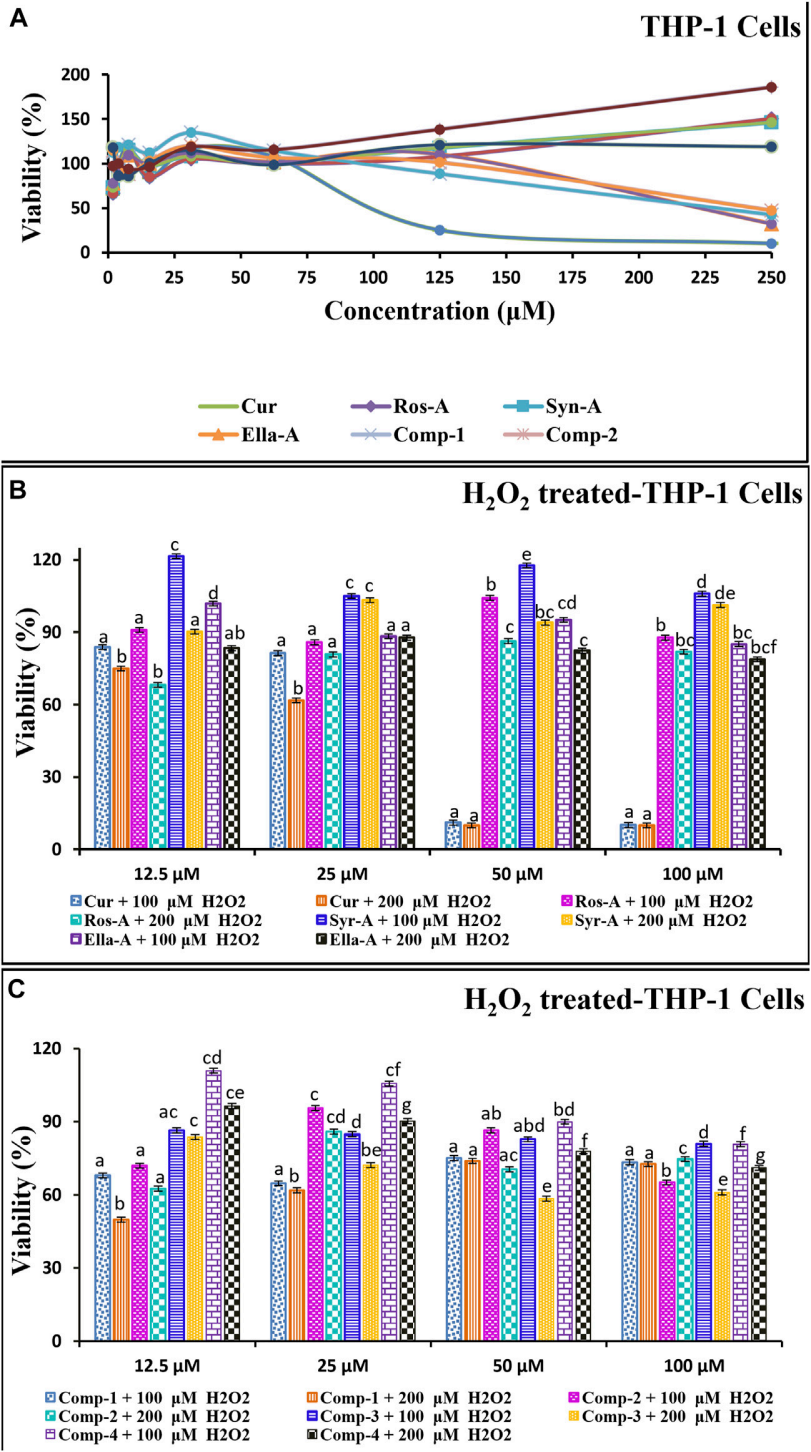
The *in vitro* cytotoxic/Cytoprotective effect of natural molecules and modes of compositions on the monocytes THP-1 Cells as well as against on H_2_O_2_-induced cytotoxicity in THP-1 cells using MTT assay. The THP-1 cells (5 × 10^5^) were incubated with Molecules (Cur, Ros-A, Ella-A, Syr-A, and Syn-A) (12.5–100 μM) or Compositions (Comp-1, Comp-2, Comp-3, Comp-4) with **(B,C)** or without H_2_O_2_ (100 or 200 μM) for 48 h **(A)**. The cytotoxicity test was expressed by % of Viability and Data presented as mean ± SD (*n* ˃ 3). Different letters indicate significant differences among treatment at *p* < 0.001.

In this section, we investigated the cytotoxic effect to understand the cytoprotective and immunomodulatory potential of the selected natural pure molecules: Cur, Ella-A, Ros-A, and Syr-A against the THP-1 cells alone as well as toward H_2_O_2_-induced cytotoxicity in these cells (Figure 1). Moreover, the potential pharmacological interaction using different formulations of these molecules was evaluated similarly for the individual compounds.

The tested comps did not show any major reduction in the monocyte’s viability and/or in H_2_O_2_-induced cytotoxicity in the monocytes THP-1. The % of viability in all treatments, using the four natural molecules alone or in the combination formulations (comps), against the THP-1 cell line showed a high peak viability value compared to the negative control (DMSO), and all treatments were significantly (*p* < 0.001) less cytotoxic than the normal control (Cells treated with H_2_O_2_). Moreover, as shown in Figure 1, the dose-response curve demonstrated that the tested molecules induced the proliferation of THP-1 cells in a dose-dependent manner (Figure 1A). Moreover, this was confirmed by the results indicated in Figure 1B showing the cytotoxicity effect of the tested formulations and the results of the H_2_O_2_-induced cytotoxicity (Figure 1C). Indeed, for Ella-A, at 12.5 μM, the percentage of viability was 102.3 ± 0.06 and 101.14 ± 0.15 and 83.43 ± 0.02, 88.38 ± 0.01 allowing the calculation of inhibitions values on THP-1 cells and H_2_O_2_-treated THP-1 cells, 100 and 200 μM, respectively. This result shows no significant difference between the viability of THP-1 alone or treated by H_2_O_2,_ showing the potent power of Ella-A cytoprotection even under H_2_O_2_ monocytes stressed. As well, for Comp-4, the viability of cells was more than 97% for a low concentration of 1.95 μM in THP-1 cells alone and more than 110% and more than 96%, allowing the calculation inhibition values on H_2_O_2_-treated THP-1 cells, 100], and 200 μM, respectively. Furthermore, both Comp-4 and Ella-A compounds have been shown to have an almost similar viability profile against the tested cell line. However, the THP-1 cell line was more sensitive to Comp-4 (97% in 1.95 μM) than Ella-A (97% in 3.90 μM). Treatment of THP-1 by 12.5 μM Ella-A or Comp-4 increased viability by at least 83% and 96% and 101% and 110%, respectively, on THP-1cells subjected to 100 and 200 μM H_2_O_2_-cytotoxicity.

### 3.5 The effects of the natural compounds on oxidative stress and the antioxidant enzymes activities in H_2_O_2_-stressed human monocytes cells

As argued before, Comp-4 and Ella-A gave very satisfactory results in terms of antioxidant activity and showed no cytotoxic effect against THP-1 cells. Therefore, Comp-4 and Ella were chosen to conduct a study at the molecular level, aiming to evaluate the effect of these two compositions on H_2_O_2_-stressed THP-1 cells using the concentrations obtained in the cytotoxicity test.

**FIGURE 2:**
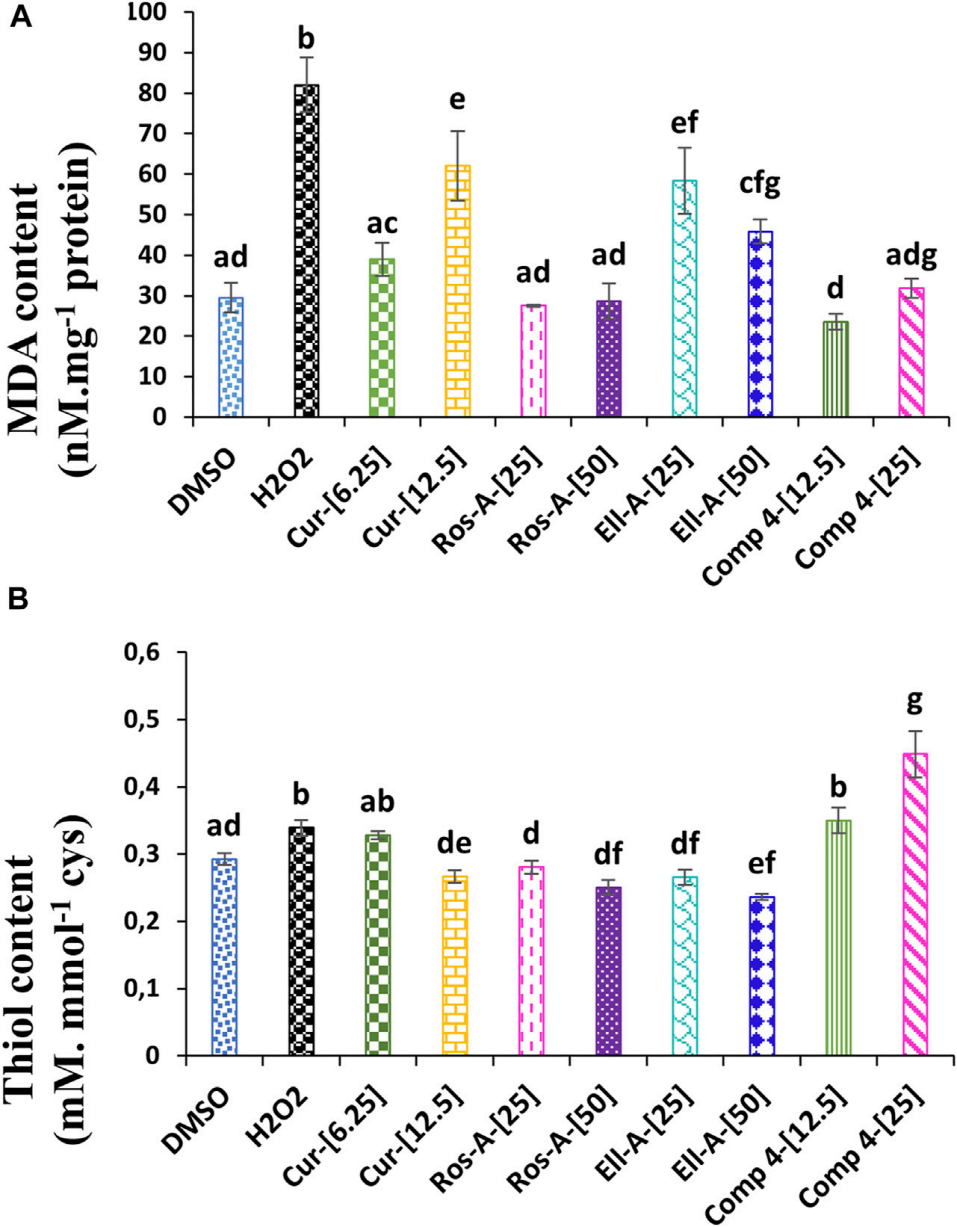
Effect of Cur, ROS-A, Ella-A and Comp-4, in THP-1 cells treated with H_2_O_2_, on **(A)** MDA content and **(B)** thiol content. Each value represents the mean of three replicate. Bars represent standard error. Different letters indicate significant differences among treatment at *p* < 0.05.

Oxidative stress is a form of attack on the cell’s constituents. It manifests when reactive oxygenated and nitrogenous oxidizing species enter or develop in the cell. These species could be radicals or not. The three most well-known are the superoxide anion (O_2_ • -), the hydroxyl radical (HO •), and hydrogen peroxide (H_2_O_2_); this hydrogen peroxide, which is naturally produced by cellular metabolism, produces very toxic intracellular hydroxyl radicals in the presence of iron, but it is almost instantly neutralized by glutathione in a healthy cell. The level of H_2_O_2_ and the degree of lipid peroxidation, quantified through measuring the MDA content, are among the siginificant indicators of oxidative stress. In the present study, compared with the control, the MDA content increased almost three times in H_2_O_2_-treated cells compared to untreated cells. However, after treatment with the different natural compounds, the level of MDA decreased significantly compared to the H_2_O_2_-treated cells. Surprisingly, 6.25 μM of Cur, 25 and 50 μM of Ros-A, and 12.5 and 25 µM of Comp-4 reduced MDA to levels comparable to those observed in the untreated cells (Figure 2A).

In the same line, the treatment of THP-1 cells with H_2_O_2_ led to a slight but significant (*p* < 0.05) increase in the content of the total free thiol when compared with non-treated cells (negative control). Moreover, except for the Ella-A at 50 μM, which decreased the total free thiol content compared to the negative control, the treatment with the other individual natural compounds did not show any effect. On the other hand, the treatment with Comp-4, especially at 25 μM, significantly increased the content of the total free thiol compared to the other conditions. Indeed, at this concentration, the content of total free thiol was 32% higher than the H_2_O_2_-treated cells (Figure 2B).

To alleviate the damage that ROS might cause, several enzymes are implicated in detoxifying these toxic compounds. These enzymes may include superoxide dismutase (SOD), catalase (CAT), glutathione reductase (GR), thioredoxin reductase (TrxR), isocitrate dehydrogenase (ICDH), and glyoxalase system (Gly I, II).

**FIGURE 3:**
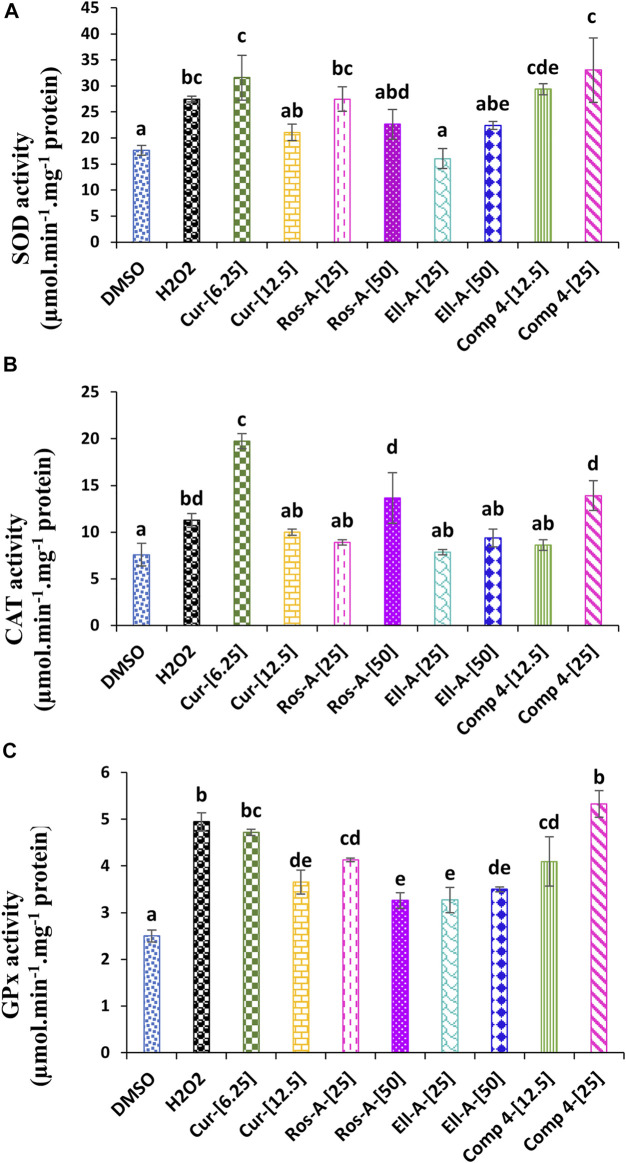
Effect of Cur, Ros-A, Ella-A and Comp-4, in THP-1 cells treated with H_2_O_2_, on **(A)** superoxide dismutase (SOD) activity, on **(B)** Catalase (CAT) activity, and **(C)** glutathione peroxidase (GPx) activity. Each value represents the mean of three replicate. Bars represent standard error. Different letters indicate significant differences among treatment at *p* < 0.05.

Superoxide dismutase activity in THP-1 cells treated with H_2_O_2_ was significantly increased compared with non-treated cells (negative control). The treatment with Cur at 6.25 μM, Ros-A at 25 μM, and Comp-4 at both concentrations were also responsible for a significant increase in the SOD activity compared to the negative control, with no significant difference if comparing the effect of these compounds with H_2_O_2_-treated cells alone (Figure 3A). The H_2_O_2_ generated by SOD can be further detoxified by catalase and glutathione peroxidase. In this study, CAT activity was increased in H_2_O_2_-stressed cells compared to the control; however, the increase was more pronounced in the cells treated with Cur at 6.25 μM, followed by Comp-4 at 25 μM, and Ros-A at 50 μM (Figure 3B). In the same line, GPx activity was also increased in cells treated with H_2_O_2_ either in the presence or not of the different natural compounds; however, the increase was the highest in cells treated with Cur at 6.25 μM and Comp-4 at 25 μM compared to the other treatments (Figure 3C).

**FIGURE 4:**
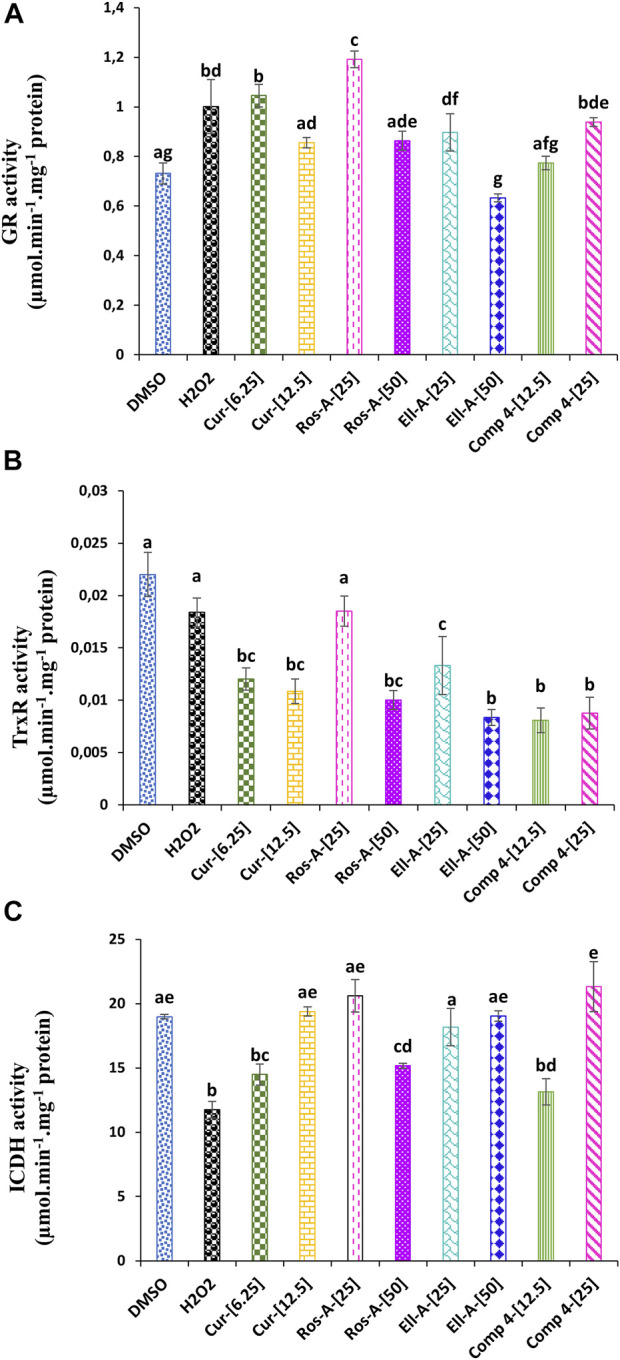
Effect of Cur, Ros-A, Ella-A and Comp 4 on glutathione reductase (GR). **(A)** Thioredoxin reductase (TrxR). **(B)** and isocitrate dehydrogenase (ICDH). **(C)** activities in THP-1 cells treated with H_2_O_2_. Each value represents the mean of three replicate. Bars represent standard error. Different letters indicate significant differences among treatment at *p* < 0.05.

Both glutathione and thioredoxin reductases were affected by H_2_O_2_. The GR activity increased compared to untreated control cells. Moreover, except for the Ella-A at 50 μM, which showed a decrease in the GR activity, the other treatments led also to a significant increase in the GR activity compared to the non-treated cells (Figure 4A). The TrxR showed a lower activity under H_2_O_2_-stress compared to the control. The treatment with molecules Cur, Ros-A at 50 μM, Ella-A, and Comp-4 led to the lowest TrxR activity (Figure 4B).

The exposure of THP-1 cells to H_2_O_2_ reduced ICDH activity (Figure 4C). However, all the natural compounds tested during this study led to the activation of this enzyme activity, especially at 12.5 μM of Cur, 25 μM of Ros-A, 50 μM of Ella-A, and 25 μM of Comp-4, showed the highest restoration of the ICDH activity and which was not significantly different from the untreated cells.

### 3.6 The effects of the natural compounds on the glyoxalase system in H_2_O_2_-stressed human monocytes cells

**FIGURE 5:**
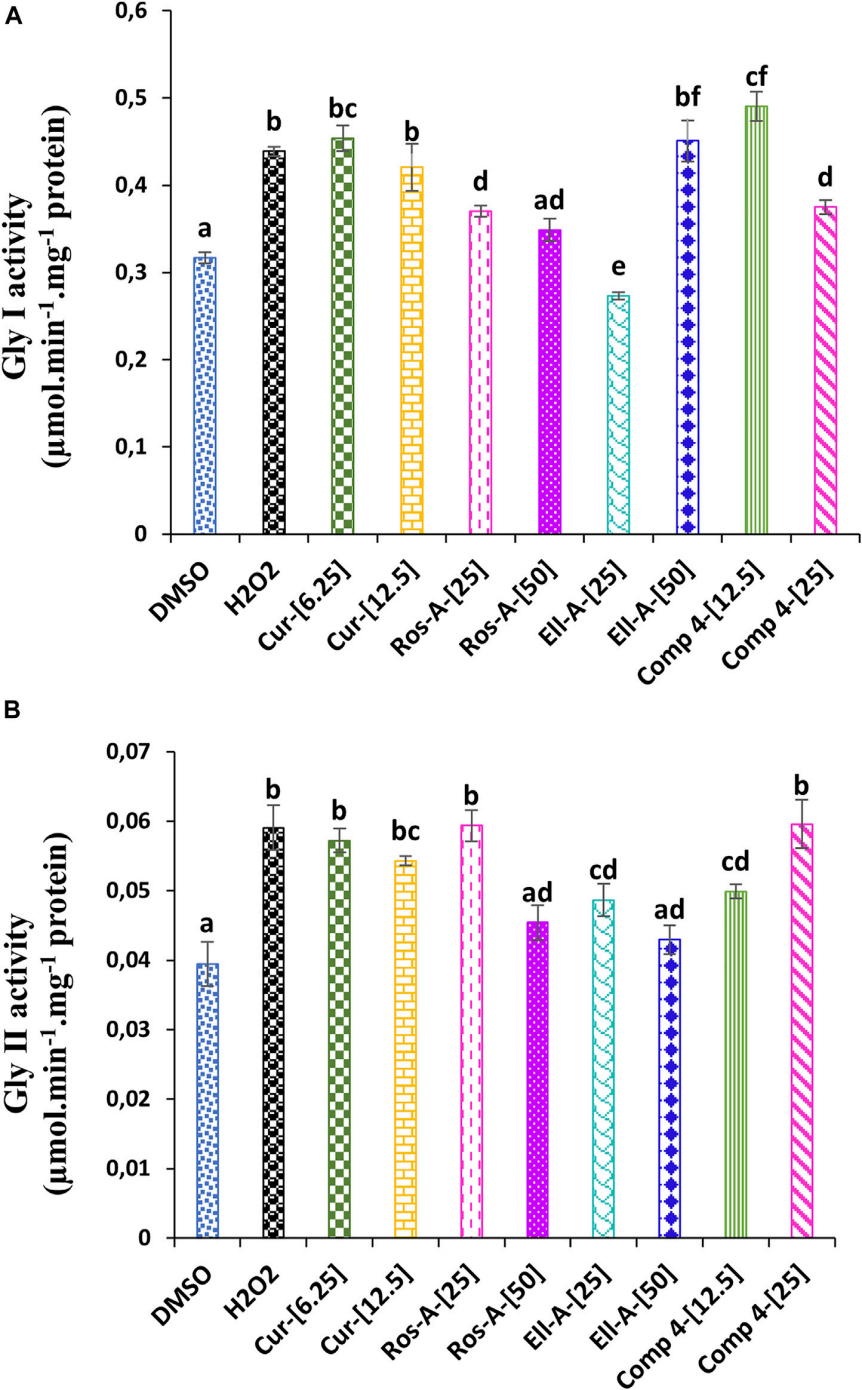
Effect of Cur, Ros-A, Ella-A and Comp 4 on glyoxalase system in THP-1 cells treated with H_2_O_2_. **(A)** Gly I activity on THP-1 treated H_2_O_2_. **(B)** Gly II activity on THP-1 treated H_2_O_2_. Each value represents the mean of three replicate. Bars represent standard error. Different letters indicate significant differences among treatment at *p* < 0.05.

As shown in Figure 4, the stress induced by H_2_O_2_ affected the glyoxalase system. H_2_O_2-_cytotoxicity increased the activity of Gly I Gly II enzymes. The addition of the different natural compounds to the culture medium was also responsible for an increase in both enzyme activities, except for Ella-A at 25 μM, which reduced the Gly I activity. Moreover, the treatment with Comp-4 at 12.5 μM led to the highest Gly I activity (Figure 5A), and the treatment with Cur, Ros-A at 25 μM, and Comp-4 at 25 μM were responsible for the highest Gly II activity (Figure 5B).

## 4 Discussion

In this study, we investigated the antioxidant activity of five natural molecules that showed, on preliminary tests, an important capacity to scavenge the DPPH and ABTS free radicals. As mentioned in Table 1, all the tested phenolic compounds exhibited significant antioxidant activity in both tests (ABTS and DPPH). However, the Ella-A and RA were the most efficient for both tests. Curcumin was also effective, but only against DPPH• free radicals. Therefore, these molecules were tested alone or in combination to assess if the synergistic action of their combination could lead to a better effect.

The multitude of optimal formulas proposed by the mixture design study led to choosing four compositions to evaluate their cytotoxicity against THP-1 cells and Ella-A, proposed as the most efficient when applied alone. From these compositions and compounds, we chose Comp-4 and Ella-A as they gave very satisfactory results in terms of antioxidant activity and showed no cytotoxic effect against THP-1 cells to conduct a study at the molecular level, aiming at evaluating the effect of these two compositions on H_2_O_2_-stressed THP-1 cells using the concentrations obtained in the cytotoxicity test. Our results clearly indicate that Ella-A or Comp-4 have potent effects on alleviating H_2_O_2_-induced cytotoxicity in the monocytes THP-1.

It is now admitted that the imbalance between the increase in ROS and the failure in the endogenous antioxidant systems are responsible for the generation of oxidative stress (Bouchmaa et al., 2018a; Xue et al., 2020). H_2_O_2_ is one of the major indicators of intracellular ROS production that can be converted, in the cell, to different other free radicals and lead to severe injury and eventually cause apoptosis (Kim et al., 2018; Liu et al., 2019). However, in healthy cells, the effect of these radicals could be counteracted by the anti-oxidative machinery. However, when their concentration increases to a certain level, these ROS are potentially capable of triggering membrane lipid peroxidation as well as damaging amino acids, proteins, nucleotides, and nucleic acids. Membrane damage resulting from lipid peroxidation produces MDA, another significant indicator of oxidative stress (Sytar et al., 2013). In our study, oxidative stress was studied in THP-1 cells, a well-known model for the intracellular generation of ROS (Reddi and Tetali, 2019; Bastin et al., 2021). As discussed above, natural compounds, selected based on their *in-vitro* anti-radical activities, were used to assess their cytoprotective effects on H_2_O_2_-induced oxidative stress in THP-1 cells. The results obtained showed that H2O2 markedly increased MDA content at 200 μM. However, the inclusion of natural compounds, especially the mixture composed of Ros-A and Cur (Comp-4), Ros-A, and 6.25 μM of Cur, suppressed MDA production in the growth medium. The antioxidant capacity of Ros-A, Cur, and Ella-A has been reported in different papers (Alagawany et al., 2017; Mansouri et al., 2020; Sharifi-Rad et al., 2020). However, this is the first report aiming to evaluate the synergistic antioxidant activity of these natural compounds in alleviating H_2_O_2_-induced stress in THP-1 cells.

Non-protein thiols play potent roles in protecting cells from ROS effects (Akinyemi et al., 2016; Oyeleye et al., 2019). Therefore, the increase in non-protein thiol contents in H_2_O_2_-treated cells (Figure 2C) could be related to the higher demand for these metabolites to scavenge free radicals. However, the treatment with Comp-4, especially at 25 μM, significantly increased the content of the total free thiol compared to the other conditions. Therefore, this increase could indicate that the effect of the combination of Ros-A and Cur (Comp-4) on the induced oxidative stress in THP-1 cells may partly be due to the increase in total free thiol compounds.

The suppression of MDA content associated with the accumulation of thiol content in Comp-4-treated cells reflects an enhanced activity of the ROS scavenging antioxidant defense system in the treated cells. Cells have evolved various mechanisms for protection against stresses. A prime example is an antioxidant system, which consists of various enzymes distributed throughout cell components and allows the dissipation of overproduced ROS. Therefore, this study suggested that protection mediated by the natural compounds against oxidative stress could be due to the activation of the cellular antioxidant defense mechanisms, including antioxidant enzymes. Indeed, in a previous study, Zhang et al. (2016) found that the anthocyanins extracted from purple vegetable roots can act to enhance the cellular antioxidant defense mechanisms through a direct radical scavenging activity and an increase in the enzyme activities of SOD, CAT, GPx, and GR, in H_2_O_2_-stressed Caco-2 cells.

Superoxide dismutase activity in THP-1 cells treated with H_2_O_2_ was significantly increased compared with non-treated cells (negative control). The treatment with Cur at 6.25 μM, Ros-A at 25 μM, and Comp-4 at both concentrations were also responsible for a significant increase in the SOD activity compared to the negative control. Previous reports have suggested that SOD is the first-line defense for scavenging toxic O_2_•− radicals and converting them to H_2_O_2_. Therefore, our study’s increase in SOD activity might be attributed to an increased O_2_•− content (Mhamdi et al., 2010). In addition to the SOD activity, the CAT and GPx activities were also increased; those enzymes act as the most H_2_O_2_ scavenging enzymes. These results indicate that the used natural compounds enhanced the O_2_•− and H_2_O_2_ scavenging enzymes and thus could be responsible for the detoxification of H_2_O_2_-induced oxidative stress in the THP-1 cells.

The glutathione- and thioredoxin-dependent systems were responsible for the intracellular antioxidant capacity. The enzymes responsible for regulating redox homeostasis, such as thiol reductases and peroxidases, depend on the pool of the reducing equivalents generated by these systems, which are GSH and thioredoxin (Ben Mrid et al., 2019; Latique et al. 2021). The addition of the natural compounds to the growth medium in the presence of H_2_O_2_ allowed an increase in the GR activity. However, a decrease in the TrxR activity was observed. The decrease in the TrxR activity is not in line with several reports that showed a decrease in the activity of this enzyme was associated with the increase in oxidative stress and thus led to apoptosis in cancer cells (Liu et al., 2018; Bouchmaa et al., 2019; Liu et al., 2019). Moreover, accumulated data reported that while the inhibition of TrxR in cancer cells may induce the excessive generation of ROS, resulting in cell death, the inhibition of this enzyme in normal cells increased the antioxidant defense and cellular proliferation (Arnér, 2020), which is the most likely effect observed in the present study.

To effectively combat ROS, the glutathione- and thioredoxin-dependent systems need enough NADPH as a cofactor (Moreno-Sánchez et al., 2017). NADP + -ICDH is one of the enzymes reported to regenerate NADPH and was therefore reported to be involved in the high resistance to oxidative stress (Moreno-Sánchez et al., 2017). In this work, except for Cur at 6.25, Ros-A at 50 μM, and Comp-4 at 12.5 μM, the different molecules increased the NADP + -ICDH activity significantly. The increase was highest under 25 μM of Comp-4 compared to the other compounds. Based on the effect of the different treatments on GR activity, the observed increase in the NADP + -ICDH activity could be responsible for providing NADPH, used as a cofactor to cope with oxidative stress through the glutathione-dependent system (Ben Mrid et al., 2019; Bouchmaa et al., 2019).

Glutathione is also vital for MG detoxification via the Gly I and Gly II enzymes and has signal properties (Sytar et al., 2013). It was reported that a high amount of MG could either act directly as a potent toxic agent affecting various cell processes or deplete GSH (Husain et al., 2010). Therefore, MG is considered an index of oxidative stress. H_2_O_2_ increased the Gly I and Gly II enzyme activities in the present study. Moreover, the different natural compounds were also responsible for increasing both enzyme activities.

Furthermore, the treatment with Comp-4 at 12.5 μM led to the highest Gly I activity, and the treatment with Cur, Ros-A at 25 μM, and Comp-4 at 25 μM were responsible for the highest Gly II activity. The increase in the enzyme activities of the glyoxalase system is correlated with the increase in GR activity, for which the pool of GSH generated is used as a co-factor for the Gly I enzyme (Ben Mrid et al., 2018); what is more, under oxidative stress or aging conditions, the activity of Gly I was reported to decrease, leading to an increase in glycation reaction and therefore, tissue injury. On the other hand, overexpression of Gly I in endothelial cells under high concentration of glucose inhibited the formation of the advanced glycation end products (AGEs), indicating the importance of this enzyme in detoxifying cells from MG, the primary precursor implicated in the formation of AGEs (Suh et al., 2018).

## 5 Conclusion

Under oxidative stress, an imbalance in the oxidant/antioxidant system occurs and leads to cell homeostasis dysregulation, characterized by excessive ROS production and reduced antioxidant protection efficiency. Natural compounds are now well known for their ability to contribute to re-establishing cell homeostasis under oxidative stress. However, these molecules are usually evaluated individually, and their synergistic effects are lacking. In the present study, an *in-vitro* cell model, the THP-1 cell line, was exposed to hydrogen peroxide to simulate a situation of oxidative stress. Different oxidative stress markers have been evaluated with or without treating the cells of natural compounds. Under stress conditions (H_2_O_2_ exposure), we increased the MDA content and ROS generation. We also observed impairment in the antioxidant defense and glyoxalase systems in the THP-1 cells. However, supplying the culture media with natural compounds increased the antioxidant defense and glyoxalase system and suppressed the ROS generation and lipid peroxidation observed in the H2O2-stressed cells. These results indicate that the natural compounds tested in this study (Cur, Ros-A, Ella-A, and Comp-4), especially Comp-4, which is composed of a mixture of Cur and Ros-A, could serve as a protectant agent to reduce H_2_O_2_ toxicity and oxidative stress in THP-1 cells. The present study also highlighted the importance of utilizing the selected molecules or Comp-4 in different fields for which antioxidant activity is sought, such as, in the food industry, as a food preservative. They may also be useful in the pharmaceutical field, either directly as antioxidants or protect against stress-induced after medical intervention or drug treatment.

## Data Availability

The original contributions presented in the study are included in the article/Supplementary Material, further inquiries can be directed to the corresponding authors.
