# Supplementary material for: Synergistic antioxidant effects of natural compounds on H2O2-induced cytotoxicity of human monocytes
Source: Front Pharmacol. 2022 Sep 1;13:830323. doi: 10.3389/fphar.2022.830323 (PMC9474927; doi:10.3389/fphar.2022.830323)
Supplement: Supplementary file 1 [file DataSheet1.docx]

Supplementary Material

# Supplementary Tables

**Supplementary-Table 1.** Simplex centroïde mixture design matrix

| Formulation | X_1_ | X_2_ | X_3_ |
| --- | --- | --- | --- |
| 1 | 100 | 0 | 0 |
| 2 | 0 | 100 | 0 |
| 3 | 0 | 0 | 100 |
| 4 | 50 | 50 | 0 |
| 5 | 50 | 0 | 50 |
| 6 | 0 | 50 | 50 |
| 7 | 33.33 | 33.33 | 33.33 |
| 8 | 66.6 | 16.66 | 16.66 |
| 9 | 16.66 | 66.6 | 16.66 |
| 10 | 16.66 | 16.66 | 66.6 |

# Supplementary Figures


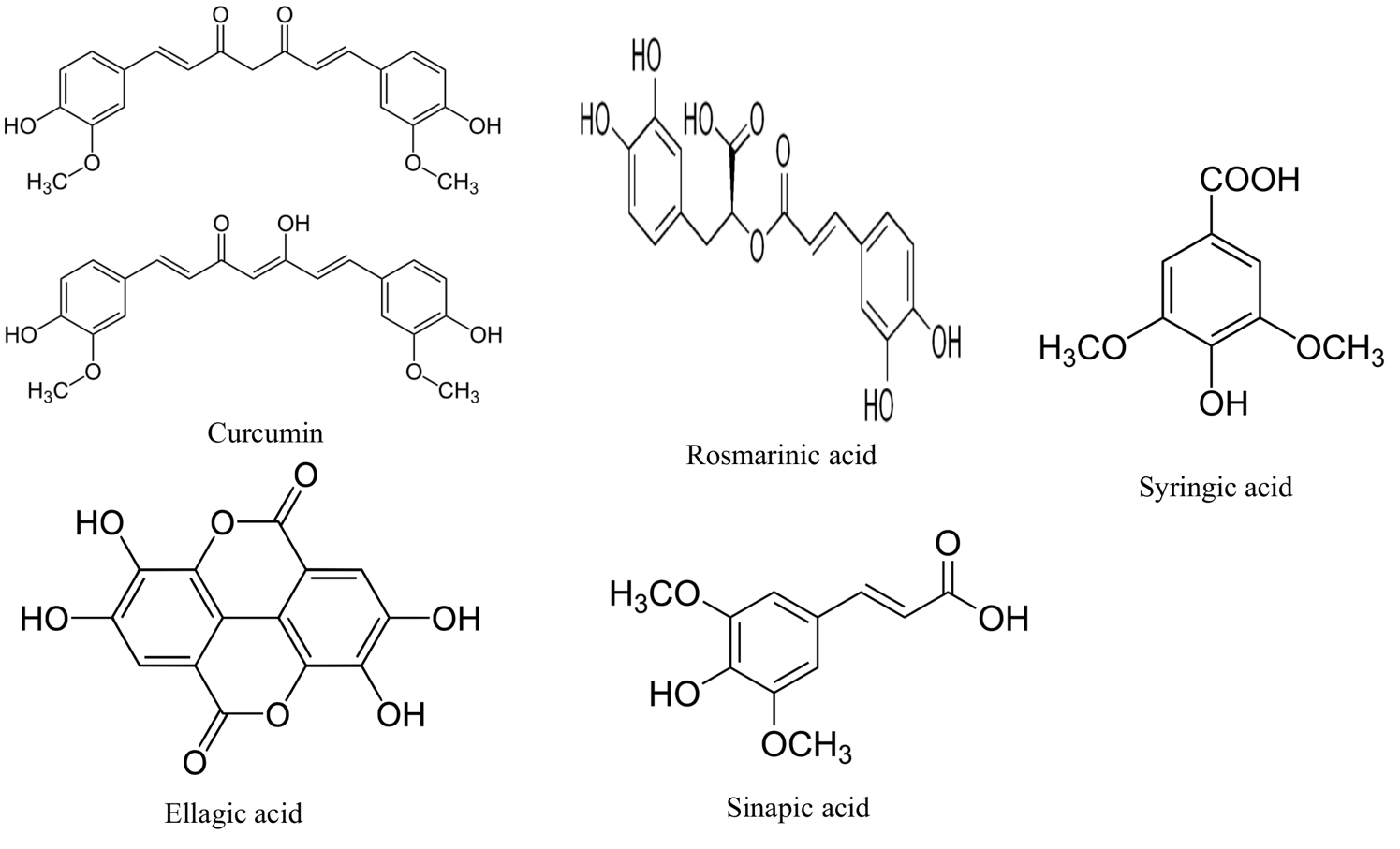


**Supplementary Figure 1.** Chemical structures of the natural compounds tested.


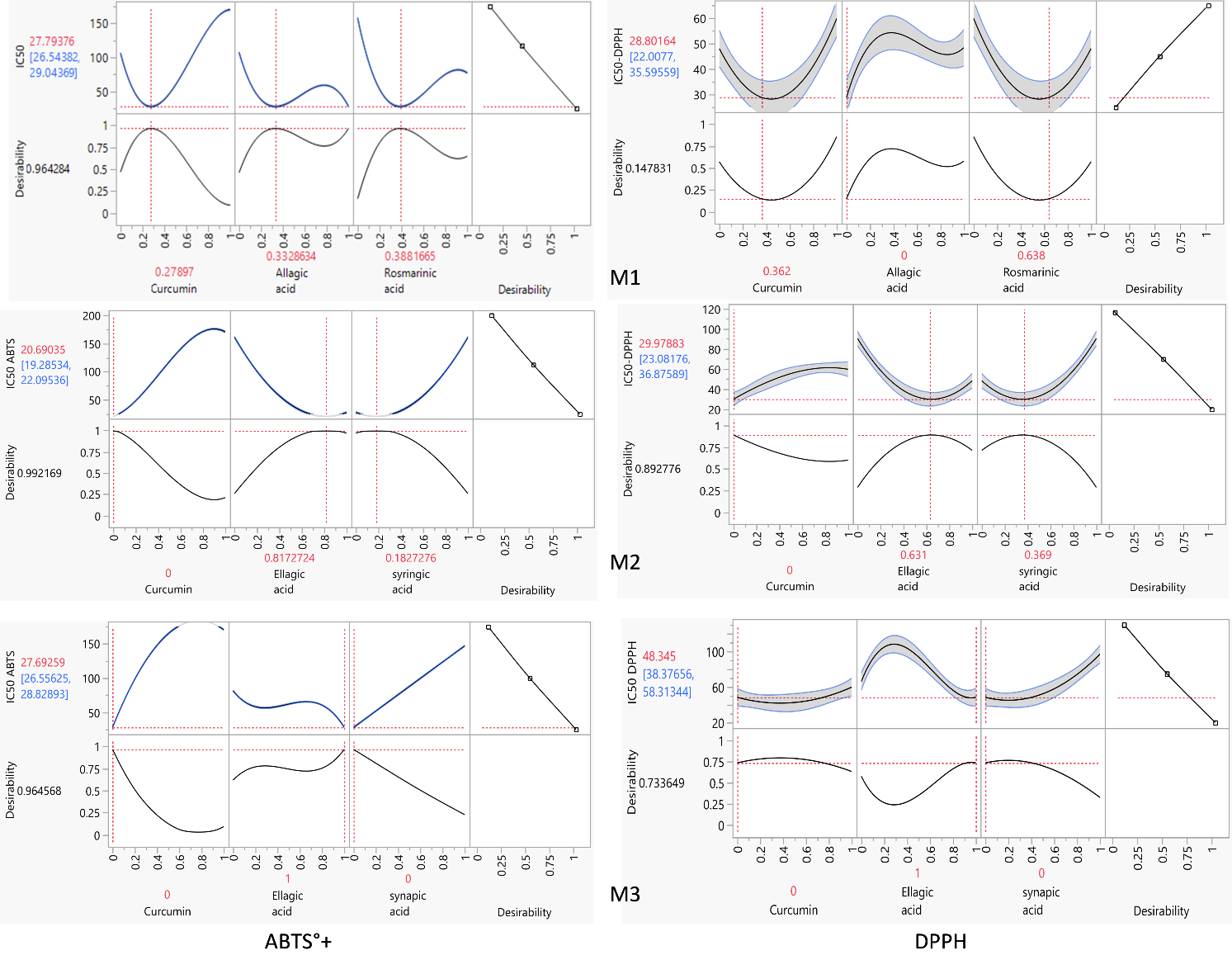


**Supplementary Figure 2.** Mixtures M1, M2 and M3 optimum profilers for ABTS°+ and DPPH tests.
